# The Effect of Silver and Samarium on the Properties of Bioglass Coatings Produced by Pulsed Laser Deposition and Spin Coating

**DOI:** 10.3390/jfb14120560

**Published:** 2023-11-28

**Authors:** Roxana Lavric, Cornelia Vreme, Cristina Busuioc, Gabriela-Olimpia Isopencu, Adrian-Ionut Nicoara, Ovidiu-Cristian Oprea, Daniel-Dumitru Banciu, Izabela Constantinoiu, Ana-Maria-Raluca Musat

**Affiliations:** 1 Department of Bioengineering and Biotechnology, Faculty of Medical Engineering, National University of Science and Technology POLITEHNICA Bucharest, RO-060042 Bucharest, Romania; roxana.lavric@stud.sim.upb.ro (R.L.);; 2 Department of Science and Engineering of Oxide Materials and Nanomaterials, Faculty of Chemical Engineering and Biotechnologies, National University of Science and Technology POLITEHNICA Bucharest, RO-060042 Bucharest, Romania; 3 Department of Chemical and Biochemical Engineering, Faculty of Chemical Engineering and Biotechnologies, National University of Science and Technology POLITEHNICA Bucharest, RO-060042 Bucharest, Romania; gabriela.isopencu@upb.ro; 4 Department of Inorganic Chemistry, Physical Chemistry and Electrochemistry, Faculty of Chemical Engineering and Biotechnologies, National University of Science and Technology POLITEHNICA Bucharest, RO-060042 Bucharest, Romania; ovidiu.oprea@upb.ro; 5 Department of Lasers, National Institute for Laser, Plasma and Radiation Physics, RO-077125 Magurele, Romania

**Keywords:** bioglass, samarium, coatings, pulsed laser deposition, spin coating, tissue engineering

## Abstract

The current study reports the use of silver (Ag) and samarium (Sm) as dopants to improve the properties of standard bioglass in terms of biological performance. This experiment considers thin films of doped bioglass obtained by pulsed laser deposition (PLD) and spin coating (SC). For both methods, some parameters were gradually varied, as the main objective was to produce a bioglass that could be used in biomedical fields. In order to study the morphology, the phase composition and other properties, the samples obtained were subjected to multiple analyses, such as thermal analysis, scanning electron microscopy (SEM), energy-dispersive X-ray spectroscopy (EDX), Fourier-transform infrared (FT-IR), Raman spectroscopy, and x-ray diffraction (XRD). Furthermore, the in vitro bioactivity of the samples, as assessed through simulated body fluid (SBF) immersion, as well as immunocytochemistry and evaluation of actin filaments, assessed through fluorescence microscopy, are reported. The results confirmed the formation of the designed vitreous target employed as the source of material in the PLD experiments only at sintering temperatures below 800 °C; this vitreous nature was preserved in the grown film as well. The presence of Ag and Ce dopants in the parent glassy matrix was validated for all stages, from powder, to target, to PLD/SC-derived coatings. Additionally, it was demonstrated that the surface topography of the layers can be adjusted by using substrates with different roughness or by modulating the processing parameters, such as substrate temperature and working pressure in PLD, rotation speed, and number of layers in SC. The developed material was found to be highly bioactive after 28 days of immersion in SBF, but it was also found to be a potential candidate for inhibiting the growth of Gram-negative bacteria and a suitable support for cell growth and proliferation.

## 1. Introduction

Biomedicine is an interdisciplinary field that focuses on the application of engineering and technology to biological and medical problems. One of the most exciting areas within this field is tissue engineering and regenerative medicine, which aims to create functional replacement tissues for patients suffering from various diseases and injuries. Recent discoveries have included the development of new techniques for generating complex tissues, such as scaffolds for new tissue regeneration, tissue adhesives, and temporary tissue barriers [1]. However, there are still significant challenges and limitations in this field. One major hurdle is the difficulty of creating tissues with the necessary complexity and functionality to be useful for implantation and repair [2]. Another challenge is the risk of immune rejection or other complications that can arise when foreign materials are introduced into the body. Researchers in this field use various biomaterials, including ceramics and polymers, with specific properties, such as biocompatibility, bioresorbability, bioavailability, and adequate physicochemical, biological, and mechanical properties, to create scaffolds that support the growth and differentiation of cells [3,4,5].

Bioglass (BG) is a type of bioactive glass that has the ability to bond with living tissues, such as bone and skin, and promote their regeneration. It was first developed in the 1960s by Dr. Larry Hench at the University of Florida, and it has since been used in a variety of medical applications such as bone grafts, dental implants, and wound healing [6]. The first BG that Dr. Hench developed was known as 45S5 and was composed of 45.0% silica, 24.5% calcium oxide, 24.5% sodium oxide, and 6.0% phosphorus pentoxide [7]. This particular composition was chosen because it closely matches the chemical composition of natural bone, making it appropriate for bone grafts and other bone-related applications. The glass composition can be further refined to control the structure and properties of bioactive glasses [8]. The most common types of BG include 45S5 Bioglass, 58S Bioglass, and 77S Bioglass. BG is commonly used in medical implants, such as bone grafts, dental implants, and cochlear implants, due to its ability to integrate with living tissue and promote bone growth [9]. It also has applications in wound healing, drug delivery, and tissue engineering [10]. However, BG has limitations, such as its brittleness and the need for specialized manufacturing processes. Its advantages include biocompatibility, osteoconductive properties, and ability to degrade over time [10].

Dopants for BGs refer to small amounts of ions or atoms that are added to the glass during manufacturing to modify its properties. They are needed to enhance the mechanical, biological, and physical properties of BGs [11]. Types of dopants for BGs include metallic ions, like copper (Cu) [12], zinc (Zn) [13], silver (Ag) [14], strontium (Sr) [15], and iron (Fe) [16] and rare-earth ions, such as cerium (Ce) [17], europium (Eu) [18], and samarium (Sm) [19]. Rare-earth dopants have been found to improve the bioactivity, osteogenic activity, and antibacterial properties of BGs [20,21]. A. Saatchi et al. [22] conducted a study on wound-healing applications using eco-friendly electrospun chitosan-based scaffolds. They tested five different ratios of cerium-doped bioactive glass to chitosan (Ce-BG/CH), ranging from 10 to 40% (*w*/*w*). Their results showed that increasing the Ce-BG/CH ratio up to 20% (*w*/*w*) improved both the degree of swelling and the mechanical properties of the scaffolds. However, exceeding this ratio decreased the beneficial effects of Ce-BG on these properties. N. Pajares et al. [23] developed a sol-gel-derived bioactive glass doped with silver (Ag) ions (Ag-BG). The resulting biomaterial can effectively combat bacterial infections while promoting bone growth. Ag-BG has been shown to inhibit bacterial growth and enhance the efficacy of conventional antibiotic treatment, as well as to increase cell proliferation and osteogenic differentiation in vitro. In vivo tests in mice demonstrated that Ag-BG microparticles induced bone regeneration, making these materials a promising therapeutic for treating resistant bacterial infections and promoting new bone formation. Moreover, Ag was integrated into several types of materials that were processed in the form of thin films by physical vapor deposition. For example, Grubova et al. [24] reported the synthesis of coatings based on silver-containing hydroxyapatite (Ag-HA)by radiofrequency magnetron sputtering, with a concentration of Ag below 0.5 wt.%, and concluded that smaller grains result in significantly higher values of nanohardness and Young’s modulus. When Ag-containing (up to 15 at.%) diamond-like carbon (DLC) films were also prepared by magnetron sputtering, the authors showed that Ag segregation plays a crucial role in determining the microstructure, mechanical, chemical and biological properties of the corresponding antibacterial samples [25]. When Ag was employed as a dopant for copper oxide (CuO) thin films grown by pulsed laser deposition (PLD), Ag nanoparticles were deposited on the surface of CuO layers that were previously prepared by laser ablation on pure copper [26].

Samarium (Sm) is a particularly important dopant for BGs, as it can enhance the ability of the glass to promote bone regeneration, making it suitable for use in bone grafts and implants. Sm-doped BGs have been shown to improve the bioactivity and biodegradation rate of BGs while also improving cell proliferation and differentiation, making them ideal for bone-tissue-engineering applications [27]. Sm-doped BGs have also been shown to possess antibacterial properties, making them promising materials for use in preventing and treating infections [28]. Furthermore, Sm-doped BGs have improved thermal stability compared to other BGs, making them less susceptible to thermal shock during manufacturing. Sm doping can also improve the mechanical properties of the glass, making it more resistant to cracking and breakage [28]. In addition, Sm-doped nanoparticles have been studied for drug-delivery and biomedical-imaging applications and the radioactive isotope Sm-153 has been used for treating different types of cancer [29].

A study by D.S. Morais et al. [30] revealed that the incorporation of Sm^3+^ in GR-HA (gelatine-reinforced hydroxyapatite) matrix led to improved surface hydrophilicity, enhanced osteoblastic cell response, and upregulated expression of relevant osteoblastic genes. Recent research investigated the potential of Sm-doped hydroxyapatite (Sm-HA) as an antimicrobial agent in biomedical applications. The studies found significant antimicrobial activity against bacterial strains such as *Escherichia coli* and *Staphylococcus aureus*, with the activity attributed to the release of Sm ions from Sm-HA materials [28,31]. X. Zhang et al. [32] studied titanium implants that were functionalized with a special type of coating made of Sm and a strontium-doped titanium dioxide (TiO_2_) nanotube array. The researchers found that this functionalization improves the ability of the implant to attach to bone tissue and also inhibits bacterial growth on the implant surface. This property is beneficial for preventing infections. I. Nica et al. investigated the biocompatibility and antibiofilm properties of Sm-doped hydroxyapatite coatings. In vitro experiments demonstrated that the coatings are biocompatible, supporting the adhesion and proliferation of bone cells. Additionally, the coatings exhibited strong antibiofilm properties, effectively inhibiting the formation and growth of biofilms on their surfaces [33]. Y. Zhang et al. [34] investigated the in vitro bioactivity and osteogenic potential of Sm-doped mesoporous bioactive glass. They found that Sm-doped glass promoted cell proliferation, osteogenic differentiation, and mineralization. It furthermore had good biocompatibility.

The BG in this study was obtained by the sol-gel method, being a quaternary high-silica bioactive glass doped with Ag and Sm [35]. Tshe ol-gel method is a well-established synthetic approach to preparing high-quality samples and consists of obtaining a sol, gelling the sol, and removing the solvent, then subjecting the material to appropriate heat treatments [36,37]. The thin films were obtained using pulsed laser deposition (PLD) and spin coating (SC). PLD is a physical vapor deposition (PVD) technique wherein a high-power pulsed laser beam is focused inside a vacuum chamber to strike a target of the material that is to be deposited. This material is vaporized from the target (in a plasma plume) and then deposited as a thin film on a substrate (such as a silicon wafer) [38]. The successful use of the PLD technique in complex stoichiometric transfer is a significant advantage and accounts for the effectiveness of PLD for depositing thin films of complex oxides. Because of its controllability, versatility, and consistency, PLD is a popular method for thin-film fabrication in optical coatings, superconducting thin films, magneto-resistive films, and other applications [39]. SC is a popular method of applying thin films to substrates, as it is highly reproducible, simple, time-efficient, and cost-effective [40]. When a material–solvent solution is spun at high speeds, the centripetal force and surface tension of the liquid together cause the solution to form an even covering. Spin coating produces a thin film that ranges in thickness from a few nanometres to a few microns after any remaining solvent has evaporated [41]. SC is used in numerous industries and technological sectors. The primary advantage of SC over other methods is its ability to produce very uniform films quickly and easily [42].

The goal of this research is to use dopants to improve the properties of a standard BG. We used Ag and Sm as dopants because, as previously stated, they have demonstrated good antibacterial properties over time. This property will likely ultimately improve the biointegration of the material. The ability of these dopants to enhance the properties of glass used to promote bone regeneration makes them suitable for use in bone grafts and implants. Sm has been shown to improve the bioactivity and biodegradation rate of BGs. Furthermore, Sm may improve the thermal stability and mechanical properties of BGs, resulting in greater resistance to cracking or breaking. As a result, we attempted to use Sm doping to produce a new type of BG for use in future medical applications.

## 2. Materials and Methods

### 2.1. Bioglass Synthesis

Quaternary high-silica bioactive glass with a composition of 65.0SiO_2_–4.5P_2_O_5_–24.0CaO–2.5Na_2_O (mol.%) doped with 1.0% Ag_2_O and 3.0% Sm_2_O_3_ (mol.%) was prepared via the sol-gel method, following the well-known steps of this procedure: (1) hydrolysis of stable solutions of metal alkoxide, with the formation of colloids or agglomerations, or dissolution of soluble salts, with the formation of clear solutions; (2) gelation based on polycondensation reactions; (3) maturation of the gel into a solid mass; (4) gel drying by thermal evaporation to obtain xerogels; and (5) calcination [43]. The main raw materials used as precursors for the oxides were tetraethyl orthosilicate (TEOS, C_8_H_20_O_4_Si, 98%, 131903, Sigma-Aldrich, Burlingtone, MA, USA), triethyl phosphate (TEP, C_6_H_15_O_4_P, ≥99%, 821141, Merck KGaA, Darmstadt, Germany), calcium nitrate tetrahydrate (Ca(NO_3_)_2_·4H_2_O, ≥99%, 102121, Merck KGaA, Darmstadt, Germany), sodium nitrite (NaNO_2_, ≥99%, 31443, Riedel-de Haën, Charlotte, NC, USA), silver nitrate (AgNO_3_, ≥99.8%, 316630, Riedel-de Haën, Charlotte, NC, USA), and samarium(III) nitrate hexahydrate (Sm(NO_3_)_3_·6H_2_O, ≥99.9%, 298123, Sigma-Aldrich, Burlingtone, MA, USA). Distilled water and ethanol (C_2_H_6_O, ≥99.8%, 32221, Sigma-Aldrich, Burlingtone, MA, USA) were used as solvents, and nitric acid (HNO_3_, ≥65%, 30709, Honeywell, Charlotte, NC, USA) was added to catalyse the hydrolysis reactions. Each preparation was planned to yield 10 g of bioactive glass. Two separate aqueous solutions in 1:3 ratios of precursor to solvent were initially prepared: one for TEOS in ethanol and one for TEP in distilled water. These solutions were prepared under magnetic stirring for 1 h until they were transparent. To adjust the pH of the TEOS solution (1.5–2 pH), several drops of HNO_3_ were used. The solution of the network modifiers was prepared in parallel by adding the required amounts of nitrates/nitrite to 80 mL of distilled water under magnetic stirring for 1 h. The clear liquid was yellowish in colour because of the presence of the Sm precursor. Afterward preparation, the solutions were mixed together (the TEP solution was added quickly, while the TEOS one was added drop by drop, with care to avoid the solution becoming translucent or opaque) and further magnetically stirred for 1 h until a homogeneous solution was formed. Afterwards, the solution was subjected to the gelation process over the next 24 h and then aged for another 48 h at room temperature. The aging step was required to ensure the formation of a three-dimensional oxide network. The xerogel was obtained after the solution was dried in an oven at 80 °C for 24 h.

### 2.2. Target Fabrication

A thermal analysis was necessary to identify the appropriate calcinating temperature, the temperature at which most gas-generating processes would be finished but the nucleation and growth of any crystalline phase would be impeded. Thus, the xerogel was crushed with an agate pestle and mortar into a fine powder and calcined at 600 °C for 4 h, with a heating rate of 10 °C/min. The calcined sample was granulated with a 2 wt.% PVA solution in water in a grinder and then passed five times through a sieve to obtain the desired granulation, then dried completely. The powder was then shaped with a uniaxial press into a disk with a diameter of 25 mm and thickness of approximately 7 mm at about 170 MPa. The target green body was thus obtained. A sintering temperature of 700 °C aws chosen after the test samples that were sintered at higher temperatures of 800 and 900 °C showed a degree of crystallinity that was too high. Moreover, the overall shrinkage that occurred on burning of the target was assessed at about 25%, considering both the green body and the final sintered sample.

### 2.3. Films Deposition

Using pulsed laser deposition (PLD) and a Ekspla laser (Nd:YAG laser, Ekspla NL301HT, Eskpla, Vilnius, Lithuania), thin films of BG were obtained. Certain parameters were then gradually varied (Table 1).

Using spin coating (SC) and a POLOS spin coater (POLOS SPIN150i, SPS, Jenkintown, PA, USA) thin films of BG were obtained. Certain parameters were gradually varied (Table 2).

### 2.4. Materials Characterization

#### 2.4.1. Thermal Analysis

Thermal analysis was conducted using a Netzsch STA 449 F3 Jupiter device (Netzsch Group, Selb, Germany) over a temperature range of 20–900 °C with an ambient atmospheric temperature. The experimental setup was necessary to allow identification of the appropriate calcination temperature.

#### 2.4.2. FT-IR Analysis

Fourier-transform infrared (FTIR) spectroscopy was used to study the chemical bonds. The wavenumber varied between 400 and 4000 cm^−1^, and a Thermo Scientific Nicolet iS50 spectrophotometer (Thermo Fisher Scientific, Waltham, MA, USA) was used for this purpose.

#### 2.4.3. Raman Analysis

The ordering degree in the samples was also investigated by Raman Spectroscopy, which was conducted on a Horiba Confocal LabRAM HR Evolution spectrophotometer (Horiba, Kyoto, Japan) with 633 nm laser excitation, the data being recorded between 100 and 1050 cm^−1^.

#### 2.4.4. SEM-EDX Analysis

To investigate the morphology, scanning electron microscopy (SEM) and energy-dispersive X-ray spectroscopy (EDX) techniques were used with a FEI Quanta Inspect F50 microscope (FEI Company, Hillsboro, OR, USA). The microscope featured an EDX probe from Oxford Instruments (Abingdon, Oxfordshire, UK) and was operated at 30 kV. Secondary electrons were employed for imaging, and no conductive coating was applied to the films.

#### 2.4.5. XRD Analysis

X-ray diffraction (XRD) was used to investigate the phase composition and crystal structure by employing a Shimadzu XRD 6000 diffractometer (Shimadzu Corporation, Kyoto, Japan) with Ni-filtered Cu Kα radiation (λ = 1.54 Å) and 2*θ* ranging between 10 and 60°.

#### 2.4.6. Biological Evaluation

The biological evaluation in vitro included a bioactivity assessment through SBF immersion, testing of antimicrobial activity against both Gram-negative and Gram-positive microorganisms, immunocytochemistry, and evaluation of actin filaments through fluorescence microscopy.

The capacity for mineralization in simulated body fluid (SBF) was estimated following immersion at 37 °C, for 28 days, in a testing solution prepared according to Kokubo [44]. Morphological characterization of the newly formed layer was carried out via SEM analysis.

To evaluate cell adhesion and viability, MC3T3-E1 mouse pre-osteoblasts (MC3T3-E1 Subclone 4, CRL-2593, ATCC, passage 27) were transferred into 13 mm-diameter glass slides and sampled for immunocytochemistry and actin-filament evaluation. Marienfeld cover slips were used as a control. Samples were UV-sterilized for 30 min on each side, placed in a 24-well plate, and seeded with 2 × 10^5^ cells/mL. Cells were allowed to adhere for 40 min in the incubator and then α-Minimum Essential Medium without phenol red (α-MEM, Gibco, Waltham, MA, USA), supplemented with 10% fetal bovine serum and 1% penicillin-streptomycin (Gibco), was added. Samples were maintained in an incubator at 37 °C with 5% CO_2_ for 48 h and evaluated by fluorescence microscopy. Fluorescently labelled phalloidin, a bicyclic peptide isolated from Amanita phalloides that shows selectivity for F-actin filaments, was used to assess morphology and cell adhesion to the substrate.

The Gram-negative bacteria *Escherichia coli* (DH5K strain from the collection of microorganisms of the Bioreactor Laboratory, Faculty of Chemical Engineering and Biotechnologies, National University of Science and Technology POLITEHNICA Bucharest) and the Gram-positive bacteria *Bacillus subtilis spizizenii nakamura* (ATCC 6633) were employed to test for antibacterial activity. The culture medium was nutrient agar (ROTH), a general-purpose nutrient medium used for the cultivation of nutritionally undemanding microorganisms. Its composition was as follows: 0.5% peptone, 0.3% beef extract/yeast extract, 0.5% NaCl, and 1.5% agar. The pH was adjusted to neutral (7.4) at 25 °C. The culture medium was sterilized by autoclaving at 121 °C for 20 min and then poured into Petri dishes. The plates were inoculated with 0.1 mL of bacterial suspension (the optical density at 600 nm of inoculum was 0.625 for *E. coli* and 0.452 for *B. subtilis*) by depletion inoculation. The plates were left for about 1 h in an oven with controlled humidity so that the bacterial suspension was uniformly impregnated in the medium and there was no excess liquid on the plate. The samples were sterilized under UV (256 nm, Portable UV lamp ROTH type IV 254/366 nm, Carl Roth, Karlsruhe, Germany) for 30 min. They were aseptically placed on the surface of the medium in the Petri dishes. Finally, the samples were incubated for 24 h at 37 °C.

## 3. Results and Discussion

### 3.1. Gel Investigation

**Figure 1:**
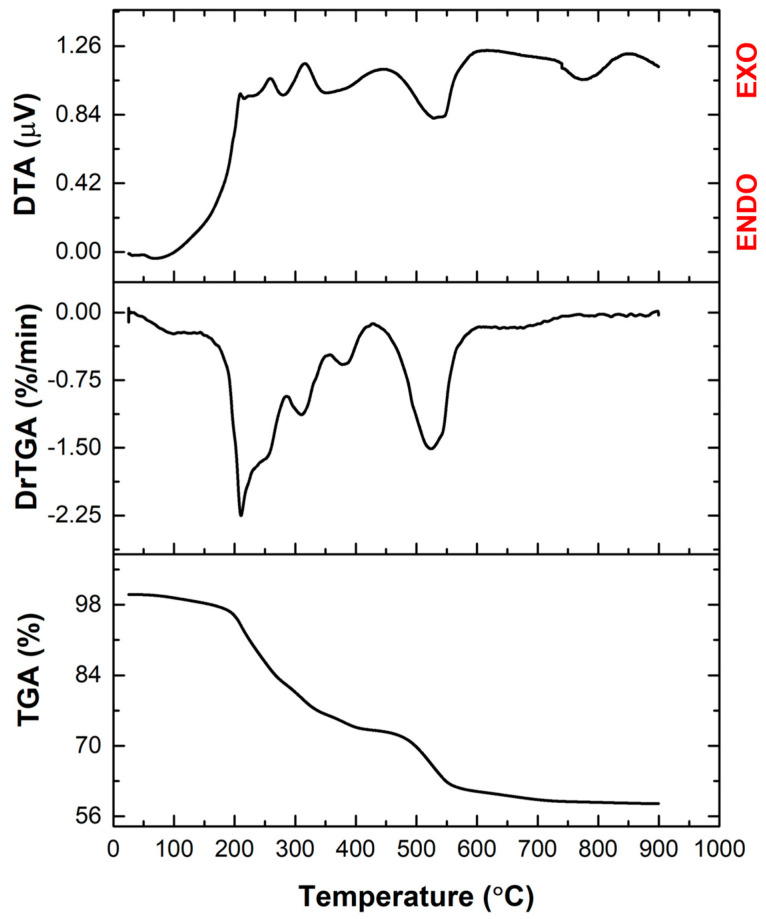
Complex thermal analysis of the dry gel. TGA—thermogravimetric analysis; DrTGA—derivative of the thermogravimetric analysis with respect to time; DTA—differential thermal analysis.

Thermal analysis was used to measure the change in the mass of dried BG gel at temperatures up to 900 °C. As can be seen in Figure 1, the TGA curve shows a total of 41.3% weight loss in two main stages, centred at around 200 and 530 °C, with the first stage being complex and containing four substages. The first stage occurs in the temperature range of 150–400 °C and is associated with the volatilization of the residual solvents and dehydration of hydrated nitrate recrystallized from the solution. The second stage, which occurs between 450–600 °C, is accompanied by endothermic effects and is associated with the decomposition of the nitrates introduced into the system. When the temperature peaked, around 40% of the sample was lost overall, but the shape of the curves showed a tendency towards thermal stabilization above 650 °C. A temperature of 600 °C was determined to be adequate for calcination, as below this value, almost all the gas-generating components are eliminated.

**Figure 2:**
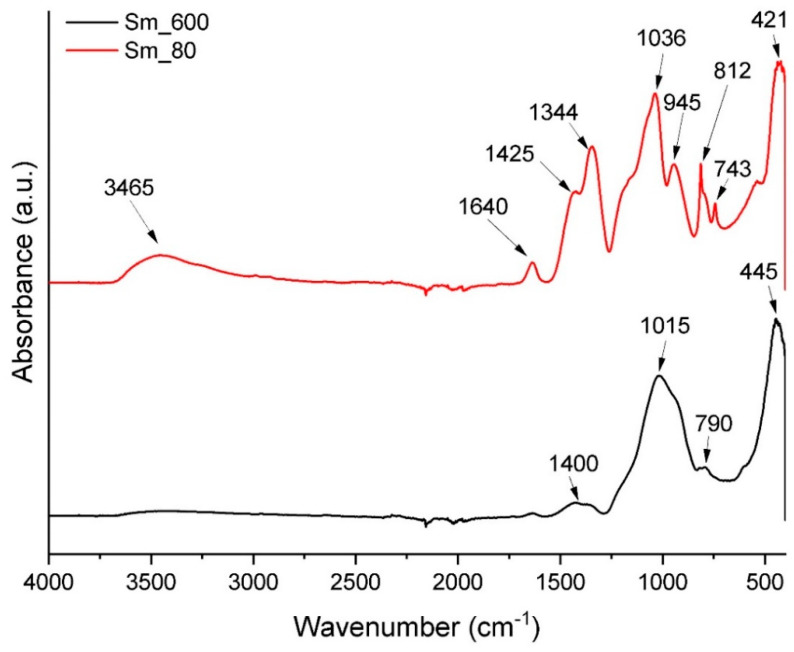
FT-IR spectra of BG gel dried at 80 °C (Sm_80) and powder calcined at 600 °C (Sm_600).

Following the thermal analysis, the xerogel and the powder calcined at 600 °C were analysed through Fourier-transform infrared (FT-IR) spectroscopy to visualize the differences between the bonds in these two samples. As can be seen in Figure 2, the FTIR spectrum for xerogel seems to be more complex than that for calcined powder. The broad absorption band with a maximum of 3465 cm^−1^ confirms the existence of hydroxyl groups [45] in the xerogel. The following 1640, 1425, and 1344 cm^−1^ maxima also correspond to hydroxylated compounds, which shows that the xerogel is not completely dried. The peak numbers of 1036 and 945 cm^−1^ most probably correspond to the presence of silicate and phosphate bonds, respectively. As can be seen in the spectrum of the powder calcined at 600 °C, the absorption bands characteristic of hydrated species are not present anymore; only the absorption maxima for silicate and phosphate ions are present in the spectrum.

### 3.2. Powder and Target Investigation

**Figure 3:**
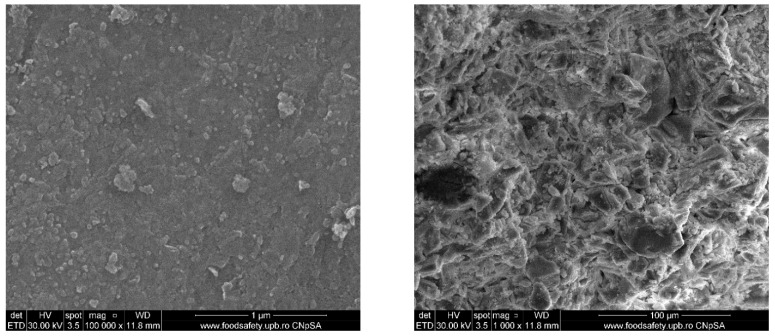
SEM images of BG powder calcined at 600 °C (**left**) and target sintered at 700 °C (**right**).

The SEM image of the powder (Figure 3) shows an extremely densely packed surface, resembling ceramic, with particle dimensions between 10 and 100 nm. Although it is desired for the target to be as compact as possible, in the SEM image of the fracture, a surface containing pores can be observed due to the low sintering temperature. Additionally, the grains, although observable, are not well-defined; it is not possible to see the boundary between them clearly. The porosity is thus interconnected due to the formation of many pore channels. Because the target should be highly compact, this being an essential requirement for correct deposition by PLD, this outcome can be considered a compromise between crystallization and densification.

**Figure 4:**
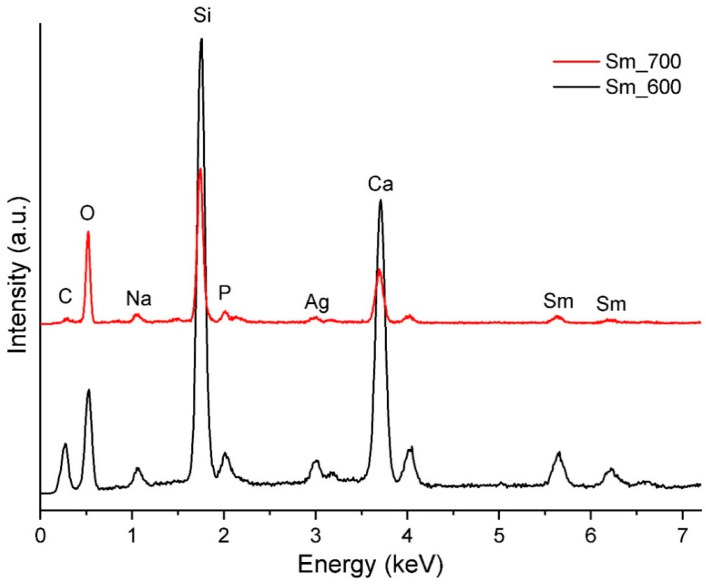
EDX spectra of BG powder calcined at 600 °C (Sm_600) and target sintered at 700 °C (Sm_700).

The BG calcined powder and sintered target were analysed through EDX to visualize the elemental composition of each. Figure 4 shows that, as expected, both samples contain the following chemical elements: Si, P, Ca, Na, Ag, Sm, O, and C. The only difference between glass powder and target is the intensity of the lines, glass powder having a higher intensity. This difference is especially visible in the lines corresponding to silicon and calcium.

**Figure 5:**
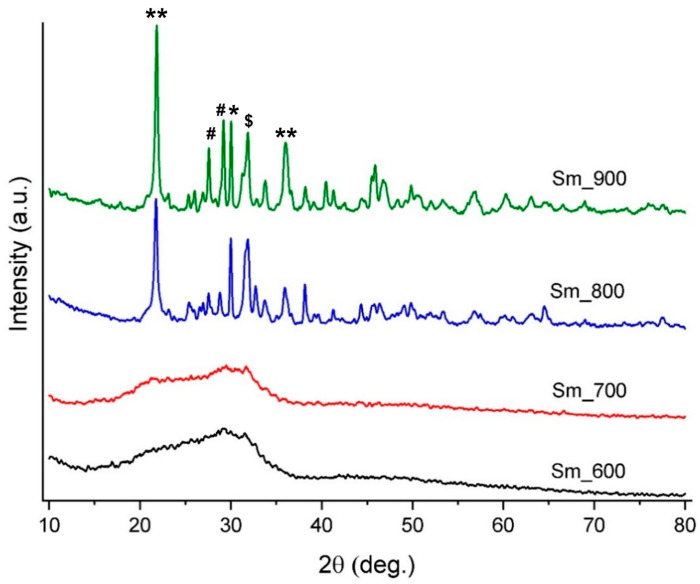
XRD patterns of BG targets sintered at different temperatures (600, 700, 800 and 900 °C). **—SiO2; *—CaSiO_3_; $—Ca_5_(PO_4_)_2_SiO_4_; #—Sm_2_O_3_.

The crystallinity of the BG powder was compared at different temperatures after the calcinating and sintering steps (Figure 5). The samples at 600 and 700 °C show an amorphous character, with very short and broad peaks. As the sintering temperature increased (from 700 to 800 °C), the samples changed from the amorphous phase to crystalline. The most crystalline sample seems to be the sample characterized after exposure to a temperature of 900 °C, with very high and sharp peaks. After analysing the phase composition, several crystalline compounds were identified, as follows: silicon dioxide (SiO_2_, ICDD 00-082-0512), wollastonite (CaSiO_3_, ICDD 00-084-0655), silicocarnotite (Ca_5_(PO_4_)_2_SiO_4_, ICDD 00-073-1181), and samarium oxide (Sm_2_O_3_, 00-076-0601), showing that the material evolves towards a vitroceramic, with multiple crystalline phases embedded in a glassy matrix.

### 3.3. PLD Films Investigation

**Figure 6:**
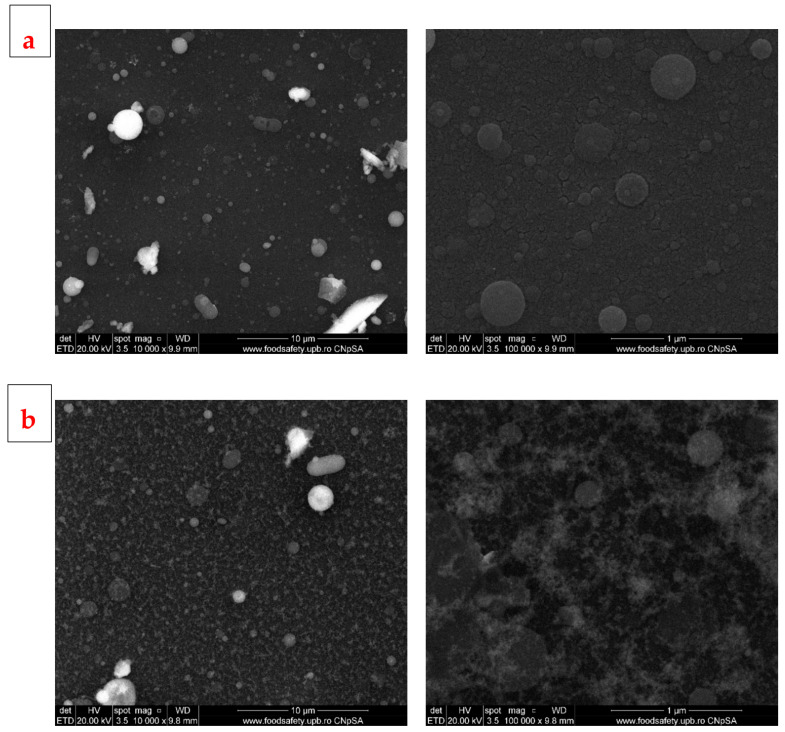
SEM images of BG films obtained by PLD at room temperature and different oxygen pressures: (**a**) 100 mTorr (PLD_RT_100) and (**b**) 700 mTorr (PLD_RT_700).

**Figure 7:**
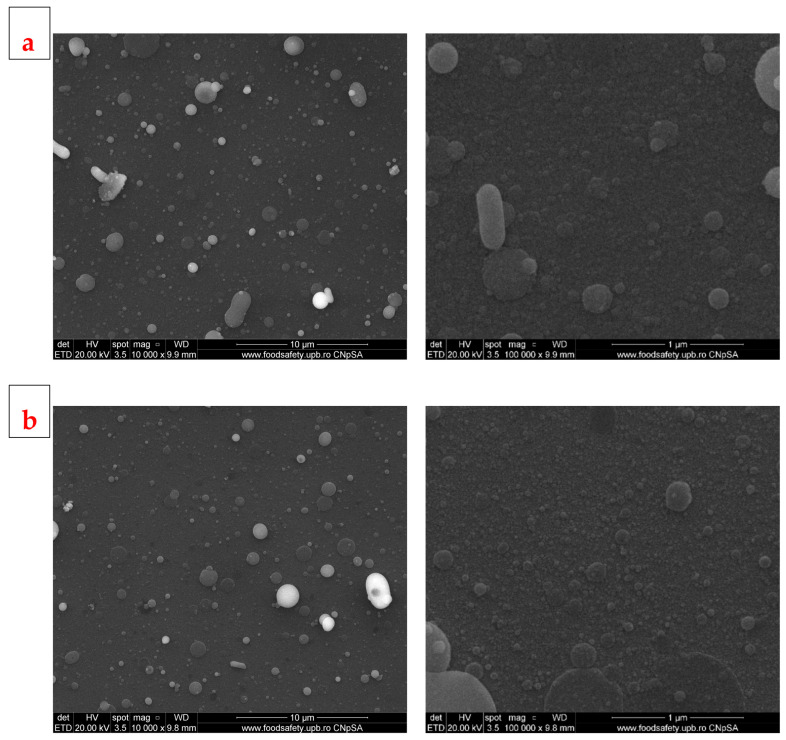
SEM images of BG films obtained by PLD at 400 °C and different oxygen pressures: (**a**) 100 mTorr (PLD_400_100) and (**b**) 700 mTorr (PLD_400_700).

The films obtained by PLD at room temperature (RT) seem to have relatively smooth surfaces, with typical droplet shapes at small magnifications and quasi-spherical particles of different sizes, raging between few hundred nm to few µm (Figure 6). Films obtained at RT and at higher magnifications show relatively round particles on the surface, but the film deposited at lower pressure has a surface full of cracks of different sizes, forming a continuous network over the entire surface, while the film deposited at higher pressure has the entire surface covered by a semi-opaque coating. In regard to the film obtained at 400 °C, both samples have rougher surfaces compared to those obtained at RT, are almost completely covered by deposited particles of different sizes, but without cracks (Figure 7). The aspects highlighted above support the idea of an influence of temperature on the development of the deposited films: a high temperature, in our case 400 °C, favours granular growth over nucleation, while at a lower temperature, nucleation will be favoured and more nuclei will appear, but will develop less, resulting in smaller grains. The other vital factor acting on the growth of deposited films is pressure: high pressure induces instability in the deposition chamber due to multiple collisions, causing the formation of a cloud-like layer on the surface and thus affecting film texture. As stated in the literature [46], these properties of the substrate, like microroughness, could have a positive influence on cell proliferation. Based on our previous research, which we performed on similar materials [47], we estimate the layer thickness to be about 50 nm for a deposition time of 30 min, as in the case of these samples. The thickness of the developed films is less important when it comes to cell interactions (for which the roughness is decisive) and of medium importance in the process of mineralization. However, the deposited coatings are continuous, and that feature, in combination with a thickness of tens of nm, provides a good surface for interaction with the surrounding body fluids.

**Figure 8:**
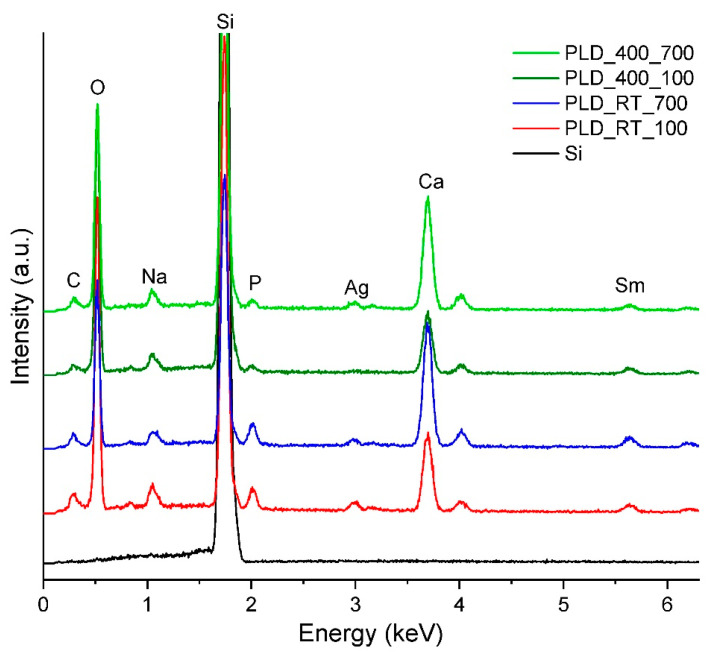
EDX spectra of BG films obtained by PLD under different experimental conditions.

To obtain the elemental composition of the films obtained by PLD, the four samples were analysed through EDX. In addition, a sample of silicon was used as a reference for comparison with the four experimental samples. As shown in Figure 8, all the samples contain the elements that were expected (Si, P, Ca, Na, Ag, Sm, and O). The main difference between the samples is the intensity of the lines; a higher intensity is visible for PLD_RT_100 and PLD_400_100, probably due to their greater thickness, which may be a result of the lower oxygen pressure.

**Figure 9:**
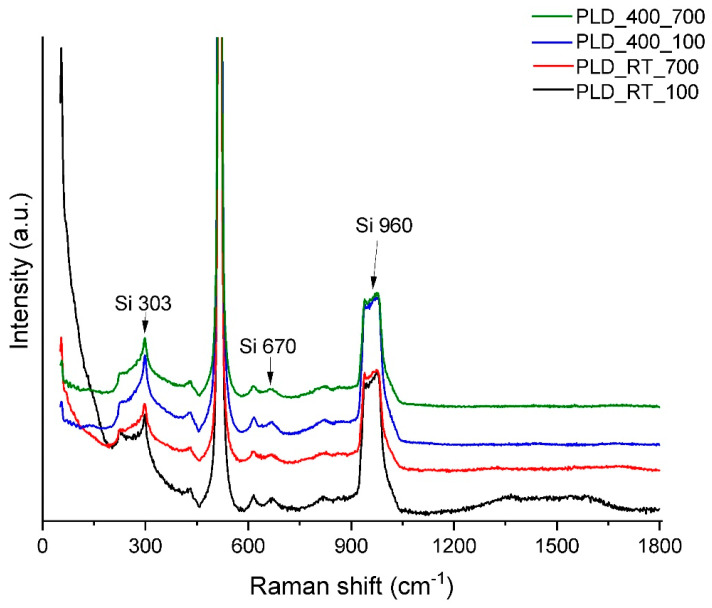
RAMAN spectra of BG films obtained by PLD under different experimental conditions.

It is quite difficult to evaluate thin films by XRD technique because of the grazing incidence working mode, as a consequence the samples obtained by PLD were analysed through Raman spectroscopy, which is known to be sensitive to ordering of chemical elements. The results show that all the bands on the spectra are attributable to the substrate [48]. There are no additional observable bands in the spectra; however, if there is ordering, it is present to a low extent (Figure 9).

### 3.4. SC Films Investigation

**Figure 10:**
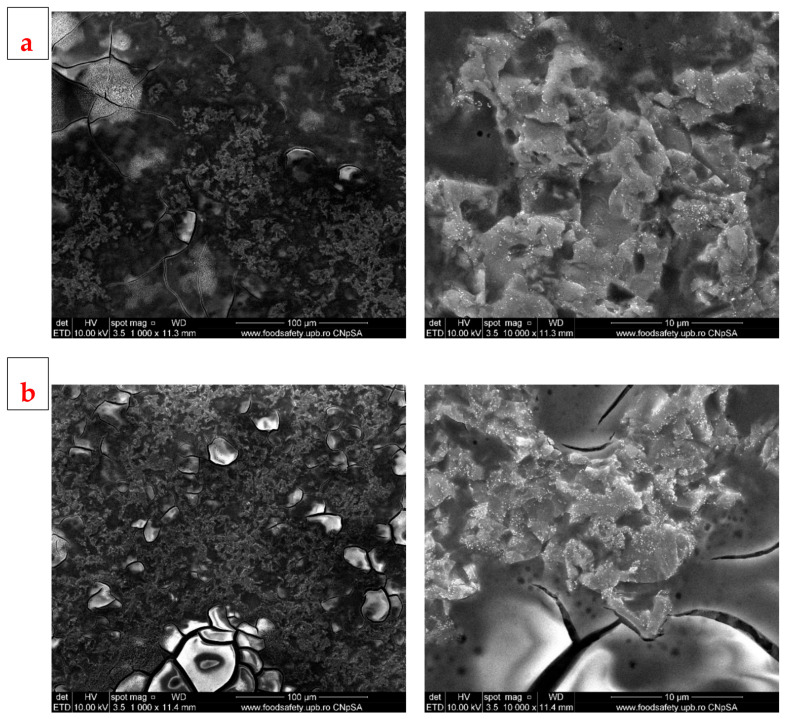
SEM images of BG films obtained by SC at (**a**) 5000 rpm (SC_3_5000) and (**b**) 10,000 rpm (SC_5_10,000).

The SEM images of the SC samples (Figure 10) highlight the very thin layer of deposited film, which fails to completely cover the Si substrate. Although a better film with fuller coverage was obtained at 5000 rpm, the analysis of both samples indicates discontinuous, highly cracked film surfaces that are full of small inclusions.

**Figure 11:**
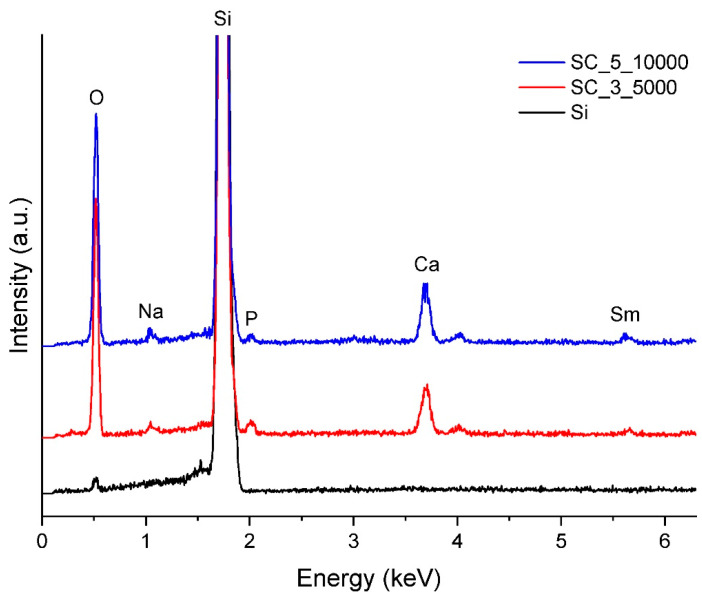
EDX spectra of BG films obtained by SC under different experimental conditions.

The samples obtained by SC were then analysed through EDX and compared with an uncovered sample of Si. A the line corresponding to silicon is present at very high intensity for both SC_3_5000 and SC_5_10,000, but the results show that the samples also contain P, Ca, Na, Sm, and O (Figure 11). Ag is not observable in the EDX spectra.

### 3.5. Biological Evaluation

**Figure 12:**
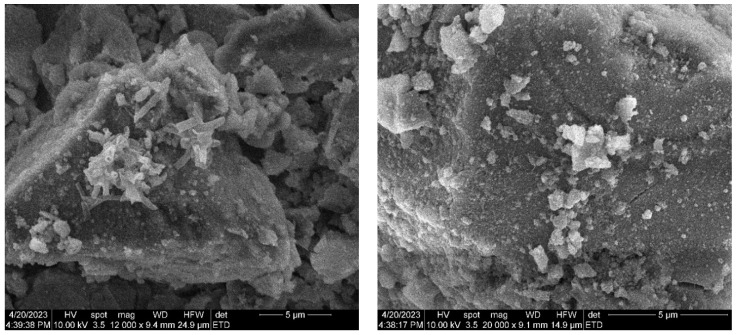

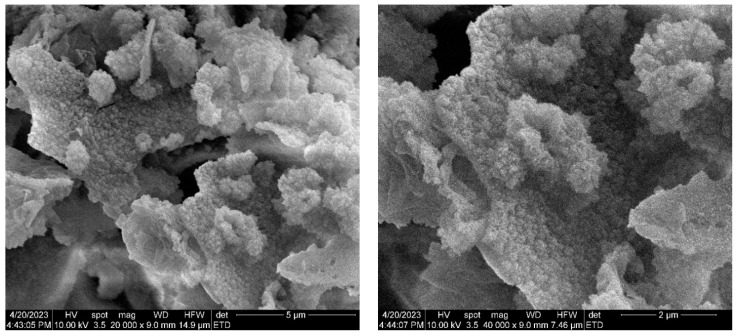
SEM images of the BG target surface (different areas and magnifications) after 28 days of immersion in SBF.

The changes to the target surfaces that occurred during SBF immersion are presented in Figure 12. The samples were involved in a biomineralization process, as demonstrated by the presence of a new surface layer with different morphological characteristics. The intensive ionic exchanges between the scaffold and the SBF resulted in the newly produced mineral structures, which are generated as a discontinuous, fluffy network of tiny raspberry-like structures, thin needles, and laminae [49,50]. The thinness and non-planarity of this apatite coating make a secondary compositional analysis challenging. However, apatite formation is undeniable because the change occurred after SBF immersion and because the surface of the original sample is covered almost entirely with novel structures.

The potential of samarium oxide (Sm_2_O_3_) nanoparticles as an antibacterial agent has already been demonstrated; Zahmatkesh et al. [51] synthesized such a material by an eco-friendly method, using curcumin as a reducing agent. The resulting nanoparticles displayed significant antibacterial activity against *Pseudomonas aeruginosa* and *Staphylococcus aureus*. Sm was also employed as a dopant for biphasic calcium phosphate (BCP) ceramics prepared via a two-step process involving sol-gel and sintering at 1200 °C. Their antibacterial activity was confirmed using the Gram-negative bacterium *Escherichia coli* and the Gram-positive bacterium *Staphylococcus aureus* [52]. Nica et al. [33] proposed novel antibacterial coatings based on hydroxyapatite nanoparticles doped with Sm, which were obtained through the dip-coating method. These nanoparticles exhibited considerable antibiofilm activity and good biocompatibility in relation to human gingival fibroblasts. Moreover, Sm concentration seems to play an important role in the antimicrobial properties these materials [31].

Thus, taking into account the fact that the developed BG contains Ag and Sm ions, both of which are known as antibacterial agents [25,28], the antimicrobial activity of this BG in the presence of *E. coli* and *B. subtilis* was assessed. The results were expressed as the inhibition zone (IZ, mm), the clear zone that appears around the sample containing the active substance, as well as the hallow zone (HZ, mm), the zone around the sample where the bacterial growth is diminished but not completely inhibited. In determining the zone of inhibition, normalization of the surface area of the samples was carried out. The films obtained by spin coating did not show a measurable response when tested in contact with Gram-negative and Gram-positive bacteria, most probably because of the low concentration of the active species, which we attributed to the reduced volume of the final layer. However, the target used as a material source for the PLD depositions, considered as a control in this case, yielded high values of IZ (2.33 mm with *E. coli* and 3.33 mm with *B. subtilis*) and HZ (7.66 mm with *E. coli*). Although the samples used in testing against *E. coli* were smaller than those used in testing against *B. subtilis*, for the PLD_RT_100 and PLD_400_100 films developed under the same oxygen pressure (100 mTorr) the Gram-negative microorganism is sensitive to lower Sm concentrations than the Gram-positive microorganism. This finding is supported by the IZ of 1.00 mm and HZ of 4.33 mm for PLD_RT_100 and the IZ of 0.66 mm and HZ of 3.66 mm for PLD_400_100 when these materials were tested against *E. coli*. In the case of *B. subtilis*, for the same samples, compared to the control, there was a reduction of the microbial population on the plate, and a change in the characteristics of the colonies, especially for the PLD_400_100 sample. As to the coatings deposited at 700 mTorr, the HZ was 2.00 mm for PLD_RT_700 and 2.33 mm for PLD_400_700 in testing against the Gram-positive microorganism.

The increased susceptibility of *E. coli* to reactive oxygen species (ROS) in the presence of Sm arises from the structure of the cell wall, which is more easily penetrable than that of Gram-positive bacteria. The fact that *B. subtilis* showed limited microbial growth in the presence of PLD_RT_100 and PLD_400_100, with a change in the characteristics of the colonies (specifically, the appearance of cellular agglomerations) reveals that the ROS were present, but not in a concentration that would result in complete inhibition.

**Figure 13:**
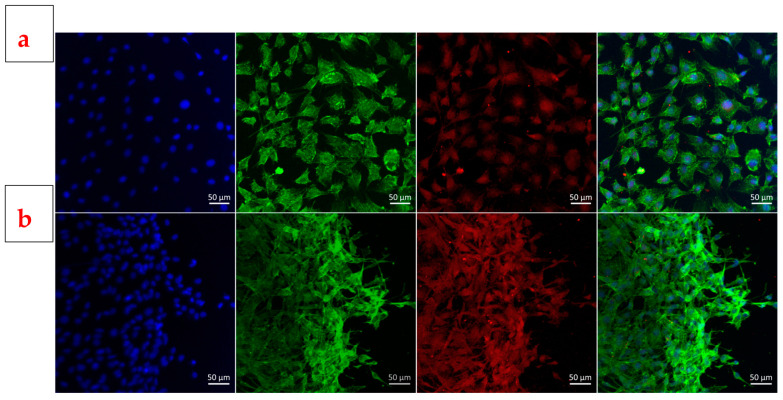
Representative fluorescence images of (**a**) cells attached to the substrate under control conditions (cells grown on cover slips) and (**b**) cell cultures on BG film obtained by PLD at 400 °C substrate temperature and 700 mTorr oxygen pressure (PLD_400_700). Nuclei are marked in blue with DAPI; actin filaments are marked in green with phalloidin; FAK is highlighted in red; and the summation image is shown at the end.

The images in the first row of Figure 13 depict the control sample, while those in the lower row show the doped BG sample (PLD_400_700 film) following biological testing. The results of fluorescence microscopy demonstrate that the cells were attached to the substrate. The main difference between these two samples seems to be the agglomeration of cells in the case of the substrate, while the cells in the control samples have a uniform distribution and appear to be fewer in number. The cells exhibit less-noticeable extensions, and there are areas of linear aggregation that indicate reduced movement on the substrate, but with focal adhesion kinase (FAK) expression present in the extensions as well. The cells have a variety of morphologies, although the majority are flattened. Adhesion kinase is found in high concentrations at the level of the cell extensions and the cell cortex. Because there are a large number of cells forming a network, it can be concluded that the substrate stimulates cell growth and proliferation. As the best biological response was achieved on the film deposited at the highest substrate temperature (400 °C) and oxygen pressure (700 mTorr), it can be concluded that surface topography plays a crucial role in cell adhesion. Specifically, the sample with the lowest proportion of droplets and the smallest droplet size, that is, the one with reduced roughness, favoured cell development to the greatest extent.

## 4. Conclusions

Thin films of silver (Ag)- and samarium (Sm)-doped bioglass (BG) were effectively produced using pulsed laser deposition (PLD) and spin coating (SC). In regard to morphology, the samples obtained by SC have some cracks on the surface, while the others have some roughness, which can aid in cell adhesion. The results of both FT-IR and Raman spectroscopy support the assertion that vitreous coatings were produced by such approaches. EDX examination reveals that dopants were present in the films, particularly in those produced by PLD. The in vitro biological assessment reveals that the selected system promotes mineralization and that cells adhered to and proliferated on the substrate due to the addition of the dopants, as it is widely known that Ag and Sm promote bioactivity and biocompatibility and have significant antibacterial capabilities. In conclusion, this work reports that the use of Ag and Sm as dopants for a typical BG can increase the biological response, allowing the materials obtained to be employed in biomedical applications.

## Figures and Tables

**Table 1:** Parameters involved in PLD experiments.

Sample	Constant Parameters	Variable Parameters	
PLD_RT_100	355 nm wavelength 62–64 mJ/pulse energy 1800 pulses 5 ns pulse duration 10 MHz frequency O_2_ atmosphere Silicon (Si) plate	Substrate temperature Pressure	Room temperature 100 mTorr
PLD_RT_700			Room temperature 700 mTorr
PLD_400_100			400 °C 100 mTorr
PLD_400_700			400 °C 700 mTorr

**Table 2:** Parameters involved in SC experiments.

Sample	Constant Parameters	Variable Parameters	
SC_3_5000	200 °C, 5 min drying 600 °C, 2 h calcining 60 s rotation time	Number of layers Rotations per minute	3 layers 5000 rpm
SC_5_10,000			5 layers 10,000 rpm

## Data Availability

Data is contained within the article.
